# Poly[[bis­[μ_2_-8-ethyl-5-oxo-2-(piperazin-1-yl)-5,8-dihydro­pyrido[2,3-*d*]pyrimidine-6-carboxyl­ato]cobalt(II)] dihydrate]

**DOI:** 10.1107/S1600536809040185

**Published:** 2009-10-10

**Authors:** Xu Qi, Ming Shao, Chen-Xin Li

**Affiliations:** a The First Affiliated Hospital, Harbin Medical University, Harbin 150001, People’s Republic of China; b The Department of Health Care Services, Harbin Medical University, Harbin 150086, People’s Republic of China

## Abstract

The title compound, {[Co(C_14_H_16_N_5_O_3_)_2_]·2H_2_O}_*n*_ or [Co(ppa)_2_]·2H_2_O}_*n*_, where ppa denotes the 8-ethyl-5-oxo-2-(piperazin-1-yl)-5,8-dihydro­pyrido[2,3-*d*]pyrimidine-6-carb­ox­yl­ate anion, was synthesized under hydro­thermal conditions. The Co^II^ atom (site symmetry 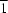) exhibits a distorted *trans*-CoN_2_O_4_ octa­hedral geometry defined by two monodentate *N*-bonded and two bidentate *O*,*O*′-bonded ppa anions. The extended two-dimensional structure is a square grid, which is consolidated by N—H⋯O hydrogen bonds. The disordered uncoordinated water mol­ecules occupy cavities within the grid.

## Related literature

For the manganese and zinc complexes of the ppa anion, see: Huang *et al.* (2008 ▶); Xu *et al.* (2009 ▶). For background to the medicinal uses of pipemidic acid, see: Mizuki *et al.* (1996 ▶).
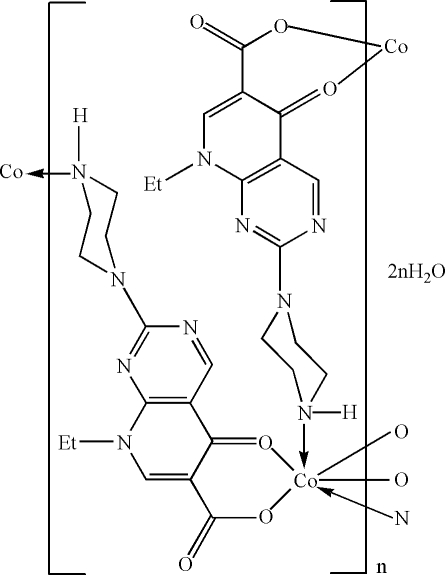


## Experimental

### 

#### Crystal data

[Co(C_14_H_16_N_5_O_3_)_2_]·2H_2_O
*M* *_r_* = 699.58
Monoclinic, 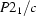

*a* = 6.1093 (3) Å
*b* = 21.3690 (11) Å
*c* = 12.5944 (6) Å
β = 101.254 (1)°
*V* = 1612.58 (14) Å^3^
*Z* = 2
Mo *K*α radiation
μ = 0.60 mm^−1^
*T* = 295 K
0.32 × 0.26 × 0.18 mm

#### Data collection

Bruker SMART CCD diffractometer
Absorption correction: multi-scan (*SADABS*; Sheldrick, 1996 ▶) *T*_min_ = 0.832, *T*_max_ = 0.900
9807 measured reflections
3894 independent reflections
3327 reflections with *I* > 2σ(*I*)
*R*_int_ = 0.029

#### Refinement

*R*[*F* ^2^ > 2σ(*F* ^2^)] = 0.060
*wR*(*F* ^2^) = 0.181
*S* = 1.12
3894 reflections
227 parameters
1 restraint
H atoms treated by a mixture of independent and constrained refinement
Δρ_max_ = 0.77 e Å^−3^
Δρ_min_ = −0.46 e Å^−3^

### 

Data collection: *SMART* (Bruker, 1998 ▶); cell refinement: *SAINT* (Bruker, 1998 ▶); data reduction: *SAINT*; program(s) used to solve structure: *SHELXS97* (Sheldrick, 2008 ▶); program(s) used to refine structure: *SHELXL97* (Sheldrick, 2008 ▶); molecular graphics: *SHELXTL* (Sheldrick, 2008 ▶); software used to prepare material for publication: *SHELXTL*.

## Supplementary Material

Crystal structure: contains datablocks I, global. DOI: 10.1107/S1600536809040185/hb5124sup1.cif

Structure factors: contains datablocks I. DOI: 10.1107/S1600536809040185/hb5124Isup2.hkl

Additional supplementary materials:  crystallographic information; 3D view; checkCIF report

## Figures and Tables

**Table 1:** Selected bond lengths (Å)

Co1—O3	2.022 (2)
Co1—O1	2.0829 (18)
Co1—N5^i^	2.265 (2)

Symmetry code: (i) 
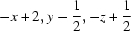
.

**Table 2:** Hydrogen-bond geometry (Å, °)

*D*—H⋯*A*	*D*—H	H⋯*A*	*D*⋯*A*	*D*—H⋯*A*
N5—H5*N*⋯O2^ii^	0.900 (10)	2.278 (14)	3.156 (4)	165 (3)

Symmetry code: (ii) 
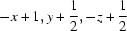
.

**Fig. 1.**
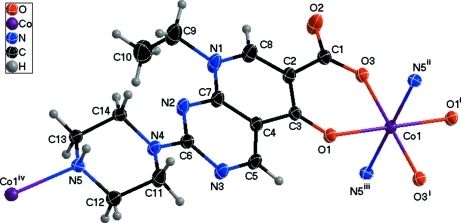
The asymmetric unit of (I) expanded to show the metal coordination and polymeric connectivity showing 50% displacement ellipsoids (water molecule O atoms have been omitted for clarity).

**Fig. 2.**
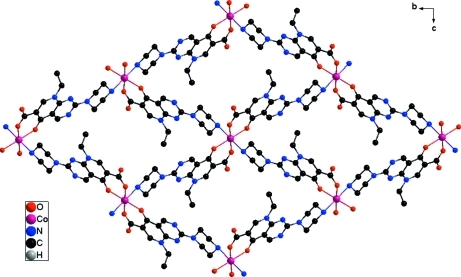
A view of part of a two-dimensional polymeric sheet in (I) showing the square-grid connectivity (H atoms and water molecule O atoms omitted for clarity).

## supplementary crystallographic information

### Comment

Pipemidic acid (Hppa, C_14_H_16_N_5_O_3_, 8-Ethyl-5,8-dihydro-5-oxo-2- (1-piperazinyl)-pyrido(2,3 - d)-pyrimidine-6-carboxylic acid) is member of a class of quinolones used to treat infections (Mizuki *et al.*, 1996). The manganese and cobalt complexes of the ppa anion have been reported (Huang *et al.*, 2008; Xu *et al.* 2009). The title cobalt(II) complex is reported here(Fig. 1).

The cobalt(II) atom is coordinated by four oxygen atoms and two N atoms from four ppa ligands (two monodentate-N and two O,*O*-bidentate) to form a square grid propagating in (Fig. 2). The disordered, uncoordinated, water molecules occupy the cavities.

### Experimental

A mixture of Co(CH_3_COO)_2_.4H_2_O (0.25 mmol), Hppa (0.5 mmol), sodium hydroxide (1 mmol) and water (12 ml) was stirred for 40 min in air. The mixture was then transferred to a 23 ml Teflon-lined hydrothermal bomb. The bomb was kept at 433 K for 96 h under autogenous pressure. Upon cooling, pink prisms of (I) were obtained from the reaction mixture.

### Refinement

The carbon-bound H atoms were positioned geometrically (C—H = 0.93–0.97 Å) and were included in the refinement in the riding model approximation, with U(H) = 1.2Ueq(C). The H on Nitrogen atoms were located in a difference Fourier map, and were refined with a distance restraint of N—H = 0.86 (1) /%A and with *U*_iso_(H) = 1.2Ueq(N).

The water H atoms could not be placed due to this disorder.

### Crystal data

**Table:**

[Co(C_14_H_16_N_5_O_3_)_2_]·2H_2_O	*F*(000) = 722
*M**_r_* = 699.58	*D*_x_ = 1.433 Mg m^−^^3^
Monoclinic, *P*2_1_/*c*	Mo *K*α radiation, λ = 0.71073 Å
Hall symbol: -P 2ybc	Cell parameters from 4362 reflections
*a* = 6.1093 (3) Å	θ = 2.5–28.2°
*b* = 21.3690 (11) Å	µ = 0.60 mm^−^^1^
*c* = 12.5944 (6) Å	*T* = 295 K
β = 101.254 (1)°	Prism, pink
*V* = 1612.58 (14) Å^3^	0.32 × 0.26 × 0.18 mm
*Z* = 2	

### Data collection

**Table:**

Bruker SMART CCD diffractometer	3894 independent reflections
Radiation source: fine-focus sealed tube	3327 reflections with *I* > 2σ(*I*)
graphite	*R*_int_ = 0.029
ω scans	θ_max_ = 28.3°, θ_min_ = 1.9°
Absorption correction: multi-scan (*SADABS*; Sheldrick, 1996)	*h* = −8→6
*T*_min_ = 0.832, *T*_max_ = 0.900	*k* = −27→28
9807 measured reflections	*l* = −15→15

### Refinement

**Table:**

Refinement on *F*^2^	Primary atom site location: structure-invariant direct methods
Least-squares matrix: full	Secondary atom site location: difference Fourier map
*R*[*F*^2^ > 2σ(*F*^2^)] = 0.060	Hydrogen site location: inferred from neighbouring sites
*wR*(*F*^2^) = 0.181	H atoms treated by a mixture of independent and constrained refinement
*S* = 1.12	*w* = 1/[σ^2^(*F*_o_^2^) + (0.0899*P*)^2^ + 1.3252*P*] where *P* = (*F*_o_^2^ + 2*F*_c_^2^)/3
3894 reflections	(Δ/σ)_max_ = 0.003
227 parameters	Δρ_max_ = 0.77 e Å^−^^3^
1 restraint	Δρ_min_ = −0.46 e Å^−^^3^

### Special details

**Table:**

Geometry. All e.s.d.'s (except the e.s.d. in the dihedral angle between two l.s. planes) are estimated using the full covariance matrix. The cell e.s.d.'s are taken into account individually in the estimation of e.s.d.'s in distances, angles and torsion angles; correlations between e.s.d.'s in cell parameters are only used when they are defined by crystal symmetry. An approximate (isotropic) treatment of cell e.s.d.'s is used for estimating e.s.d.'s involving l.s. planes.
Refinement. Refinement of *F*^2^ against ALL reflections. The weighted *R*-factor *wR* and goodness of fit *S* are based on *F*^2^, conventional *R*-factors *R* are based on *F*, with *F* set to zero for negative *F*^2^. The threshold expression of *F*^2^ > σ(*F*^2^) is used only for calculating *R*-factors(gt) *etc*. and is not relevant to the choice of reflections for refinement. *R*-factors based on *F*^2^ are statistically about twice as large as those based on *F*, and *R*- factors based on ALL data will be even larger.

### Fractional atomic coordinates and isotropic or equivalent isotropic displacement parameters (Å^2^)

**Table:**

	*x*	*y*	*z*	*U*_iso_*/*U*_eq_	Occ. (<1)
O1W	0.669 (4)	0.5177 (8)	0.5335 (9)	0.255 (11)	0.50
O2W	−0.024 (4)	0.5581 (10)	0.4291 (11)	0.254 (10)	0.50
Co1	0.5000	0.5000	0.0000	0.02469 (18)	
O1	0.6496 (3)	0.57810 (9)	0.08289 (16)	0.0324 (4)	
O2	0.1437 (5)	0.51651 (14)	0.2499 (2)	0.0626 (8)	
N1	0.5101 (5)	0.67072 (13)	0.3478 (2)	0.0487 (7)	
N2	0.7758 (5)	0.74617 (12)	0.3332 (2)	0.0390 (6)	
N3	1.0138 (5)	0.73519 (13)	0.2023 (2)	0.0437 (7)	
N4	1.0250 (4)	0.82327 (11)	0.3106 (2)	0.0362 (6)	
C1	0.2840 (5)	0.52788 (13)	0.1939 (2)	0.0328 (6)	
C2	0.4347 (5)	0.58348 (13)	0.2241 (2)	0.0330 (6)	
C3	0.6059 (4)	0.60347 (12)	0.1662 (2)	0.0275 (5)	
C4	0.7269 (5)	0.65898 (13)	0.2104 (2)	0.0318 (6)	
C5	0.9081 (5)	0.68298 (14)	0.1695 (3)	0.0398 (7)	
H5	0.9566	0.6603	0.1155	0.048*	
C6	0.9339 (5)	0.76685 (13)	0.2815 (2)	0.0334 (6)	
C7	0.6763 (5)	0.69244 (13)	0.2976 (3)	0.0364 (6)	
C8	0.3996 (6)	0.61779 (15)	0.3104 (3)	0.0452 (8)	
H8	0.2905	0.6036	0.3467	0.054*	
C9	0.4470 (8)	0.7043 (2)	0.4423 (4)	0.0646 (12)	
H9A	0.4693	0.7489	0.4351	0.078*	
H9B	0.2902	0.6972	0.4425	0.078*	
C10	0.5781 (12)	0.6828 (4)	0.5417 (5)	0.118 (2)	
H10A	0.5474	0.6393	0.5513	0.177*	
H10B	0.5417	0.7065	0.6006	0.177*	
H10C	0.7335	0.6880	0.5401	0.177*	
C11	1.1614 (6)	0.85706 (17)	0.2461 (3)	0.0478 (8)	
H11A	1.0667	0.8839	0.1943	0.057*	
H11B	1.2345	0.8274	0.2062	0.057*	
C12	1.3372 (5)	0.89675 (15)	0.3196 (3)	0.0400 (7)	
H12A	1.4448	0.8690	0.3630	0.048*	
H12B	1.4162	0.9216	0.2747	0.048*	
C13	1.1106 (5)	0.90153 (13)	0.4537 (2)	0.0330 (6)	
H13A	1.0424	0.9293	0.4989	0.040*	
H13B	1.2078	0.8731	0.5011	0.040*	
C14	0.9295 (5)	0.86418 (14)	0.3823 (3)	0.0353 (6)	
H14A	0.8505	0.8392	0.4270	0.042*	
H14B	0.8233	0.8925	0.3395	0.042*	
N5	1.2458 (4)	0.93890 (10)	0.39246 (19)	0.0286 (5)	
O3	0.3027 (3)	0.49641 (8)	0.11147 (17)	0.0295 (4)	
H5N	1.155 (5)	0.9660 (14)	0.350 (2)	0.044*	

### Atomic displacement parameters (Å^2^)

**Table:**

	*U*^11^	*U*^22^	*U*^33^	*U*^12^	*U*^13^	*U*^23^
O1W	0.52 (4)	0.180 (13)	0.073 (8)	−0.084 (17)	0.063 (13)	0.045 (8)
O2W	0.35 (2)	0.33 (2)	0.122 (10)	−0.09 (2)	0.147 (13)	−0.030 (13)
Co1	0.0276 (3)	0.0158 (3)	0.0304 (3)	−0.00028 (17)	0.0050 (2)	−0.00242 (17)
O1	0.0350 (10)	0.0243 (9)	0.0395 (11)	−0.0065 (8)	0.0113 (8)	−0.0089 (8)
O2	0.0732 (18)	0.0635 (16)	0.0631 (17)	−0.0401 (15)	0.0429 (15)	−0.0297 (14)
N1	0.0630 (18)	0.0404 (15)	0.0494 (16)	−0.0222 (14)	0.0271 (14)	−0.0189 (12)
N2	0.0494 (15)	0.0296 (12)	0.0413 (14)	−0.0138 (11)	0.0167 (11)	−0.0129 (10)
N3	0.0454 (15)	0.0348 (14)	0.0556 (16)	−0.0152 (12)	0.0221 (13)	−0.0200 (12)
N4	0.0390 (13)	0.0268 (12)	0.0462 (14)	−0.0093 (10)	0.0169 (11)	−0.0132 (10)
C1	0.0354 (14)	0.0250 (13)	0.0379 (15)	−0.0061 (11)	0.0068 (12)	−0.0030 (11)
C2	0.0409 (15)	0.0244 (13)	0.0343 (14)	−0.0074 (11)	0.0091 (12)	−0.0035 (10)
C3	0.0309 (13)	0.0192 (12)	0.0314 (13)	−0.0023 (10)	0.0034 (10)	−0.0024 (10)
C4	0.0354 (14)	0.0241 (12)	0.0365 (14)	−0.0064 (11)	0.0082 (11)	−0.0064 (11)
C5	0.0434 (16)	0.0300 (14)	0.0498 (18)	−0.0102 (12)	0.0186 (14)	−0.0180 (13)
C6	0.0338 (14)	0.0248 (13)	0.0427 (16)	−0.0058 (11)	0.0097 (12)	−0.0087 (11)
C7	0.0449 (16)	0.0269 (13)	0.0397 (15)	−0.0099 (12)	0.0139 (13)	−0.0087 (11)
C8	0.057 (2)	0.0352 (16)	0.0486 (18)	−0.0198 (15)	0.0238 (16)	−0.0107 (14)
C9	0.080 (3)	0.058 (2)	0.065 (3)	−0.030 (2)	0.035 (2)	−0.025 (2)
C10	0.121 (5)	0.155 (7)	0.082 (4)	−0.025 (5)	0.028 (4)	−0.020 (4)
C11	0.055 (2)	0.0429 (18)	0.0501 (19)	−0.0253 (15)	0.0221 (16)	−0.0187 (15)
C12	0.0370 (15)	0.0326 (15)	0.0537 (18)	−0.0113 (12)	0.0167 (13)	−0.0110 (13)
C13	0.0334 (14)	0.0266 (13)	0.0402 (15)	−0.0067 (11)	0.0104 (12)	−0.0072 (11)
C14	0.0290 (13)	0.0276 (13)	0.0507 (17)	−0.0062 (11)	0.0114 (12)	−0.0119 (12)
N5	0.0283 (11)	0.0208 (10)	0.0362 (12)	−0.0017 (8)	0.0053 (9)	−0.0010 (9)
O3	0.0337 (10)	0.0199 (9)	0.0353 (11)	−0.0035 (7)	0.0072 (8)	−0.0029 (7)

### Geometric parameters (Å, °)

**Table:**

Co1—O3^i^	2.022 (2)	C4—C5	1.405 (4)
Co1—O3	2.022 (2)	C5—H5	0.9300
Co1—O1^i^	2.0829 (18)	C8—H8	0.9300
Co1—O1	2.0829 (18)	C9—C10	1.425 (9)
Co1—N5^ii^	2.265 (2)	C9—H9A	0.9700
Co1—N5^iii^	2.265 (2)	C9—H9B	0.9700
O1—C3	1.255 (3)	C10—H10A	0.9600
O2—C1	1.235 (4)	C10—H10B	0.9600
N1—C8	1.354 (4)	C10—H10C	0.9600
N1—C7	1.376 (4)	C11—C12	1.530 (4)
N1—C9	1.503 (5)	C11—H11A	0.9700
N2—C7	1.335 (4)	C11—H11B	0.9700
N2—C6	1.341 (4)	C12—N5	1.471 (4)
N3—C5	1.315 (4)	C12—H12A	0.9700
N3—C6	1.371 (4)	C12—H12B	0.9700
N4—C6	1.348 (3)	C13—N5	1.471 (3)
N4—C14	1.459 (3)	C13—C14	1.510 (4)
N4—C11	1.462 (4)	C13—H13A	0.9700
C1—O3	1.261 (3)	C13—H13B	0.9700
C1—C2	1.506 (4)	C14—H14A	0.9700
C2—C8	1.362 (4)	C14—H14B	0.9700
C2—C3	1.451 (4)	N5—Co1^iv^	2.265 (2)
C3—C4	1.450 (4)	N5—H5N	0.900 (10)
C4—C7	1.396 (4)		
			
O3^i^—Co1—O3	180.0	N1—C8—H8	117.2
O3^i^—Co1—O1^i^	86.86 (7)	C2—C8—H8	117.2
O3—Co1—O1^i^	93.14 (7)	C10—C9—N1	110.8 (5)
O3^i^—Co1—O1	93.14 (7)	C10—C9—H9A	109.5
O3—Co1—O1	86.86 (7)	N1—C9—H9A	109.5
O1^i^—Co1—O1	180.0	C10—C9—H9B	109.5
O3^i^—Co1—N5^ii^	90.28 (8)	N1—C9—H9B	109.5
O3—Co1—N5^ii^	89.72 (8)	H9A—C9—H9B	108.1
O1^i^—Co1—N5^ii^	91.18 (8)	C9—C10—H10A	109.5
O1—Co1—N5^ii^	88.82 (8)	C9—C10—H10B	109.5
O3^i^—Co1—N5^iii^	89.72 (8)	H10A—C10—H10B	109.5
O3—Co1—N5^iii^	90.28 (8)	C9—C10—H10C	109.5
O1^i^—Co1—N5^iii^	88.82 (8)	H10A—C10—H10C	109.5
O1—Co1—N5^iii^	91.18 (8)	H10B—C10—H10C	109.5
N5^ii^—Co1—N5^iii^	180.0	N4—C11—C12	110.3 (3)
C3—O1—Co1	128.37 (17)	N4—C11—H11A	109.6
C8—N1—C7	119.0 (3)	C12—C11—H11A	109.6
C8—N1—C9	119.1 (3)	N4—C11—H11B	109.6
C7—N1—C9	121.9 (3)	C12—C11—H11B	109.6
C7—N2—C6	116.4 (2)	H11A—C11—H11B	108.1
C5—N3—C6	115.1 (3)	N5—C12—C11	114.2 (3)
C6—N4—C14	120.7 (2)	N5—C12—H12A	108.7
C6—N4—C11	122.7 (2)	C11—C12—H12A	108.7
C14—N4—C11	112.8 (2)	N5—C12—H12B	108.7
O2—C1—O3	123.3 (3)	C11—C12—H12B	108.7
O2—C1—C2	118.0 (3)	H12A—C12—H12B	107.6
O3—C1—C2	118.6 (2)	N5—C13—C14	113.3 (2)
C8—C2—C3	118.9 (3)	N5—C13—H13A	108.9
C8—C2—C1	116.3 (3)	C14—C13—H13A	108.9
C3—C2—C1	124.8 (2)	N5—C13—H13B	108.9
O1—C3—C4	119.9 (2)	C14—C13—H13B	108.9
O1—C3—C2	125.8 (2)	H13A—C13—H13B	107.7
C4—C3—C2	114.3 (2)	N4—C14—C13	110.5 (2)
C7—C4—C5	114.4 (2)	N4—C14—H14A	109.5
C7—C4—C3	123.1 (3)	C13—C14—H14A	109.5
C5—C4—C3	122.5 (3)	N4—C14—H14B	109.5
N3—C5—C4	124.7 (3)	C13—C14—H14B	109.5
N3—C5—H5	117.7	H14A—C14—H14B	108.1
C4—C5—H5	117.7	C13—N5—C12	108.4 (2)
N2—C6—N4	117.5 (2)	C13—N5—Co1^iv^	112.89 (17)
N2—C6—N3	125.7 (3)	C12—N5—Co1^iv^	115.23 (17)
N4—C6—N3	116.9 (3)	C13—N5—H5N	108 (2)
N2—C7—N1	117.7 (3)	C12—N5—H5N	107 (2)
N2—C7—C4	123.3 (3)	Co1^iv^—N5—H5N	105 (2)
N1—C7—C4	119.0 (3)	C1—O3—Co1	135.54 (18)
N1—C8—C2	125.6 (3)		
			
O3^i^—Co1—O1—C3	−179.8 (2)	C8—N1—C7—N2	177.2 (3)
O3—Co1—O1—C3	0.2 (2)	C9—N1—C7—N2	−2.4 (5)
N5^ii^—Co1—O1—C3	−89.6 (2)	C8—N1—C7—C4	−0.5 (5)
N5^iii^—Co1—O1—C3	90.4 (2)	C9—N1—C7—C4	179.9 (4)
O2—C1—C2—C8	−1.4 (5)	C5—C4—C7—N2	6.3 (5)
O3—C1—C2—C8	176.6 (3)	C3—C4—C7—N2	−174.7 (3)
O2—C1—C2—C3	−179.3 (3)	C5—C4—C7—N1	−176.2 (3)
O3—C1—C2—C3	−1.3 (4)	C3—C4—C7—N1	2.8 (5)
Co1—O1—C3—C4	−178.49 (19)	C7—N1—C8—C2	−1.4 (6)
Co1—O1—C3—C2	−0.6 (4)	C9—N1—C8—C2	178.2 (4)
C8—C2—C3—O1	−176.7 (3)	C3—C2—C8—N1	0.9 (6)
C1—C2—C3—O1	1.2 (5)	C1—C2—C8—N1	−177.2 (3)
C8—C2—C3—C4	1.3 (4)	C8—N1—C9—C10	91.4 (5)
C1—C2—C3—C4	179.2 (3)	C7—N1—C9—C10	−89.0 (5)
O1—C3—C4—C7	175.0 (3)	C6—N4—C11—C12	−149.0 (3)
C2—C3—C4—C7	−3.1 (4)	C14—N4—C11—C12	52.8 (4)
O1—C3—C4—C5	−6.0 (4)	N4—C11—C12—N5	−53.2 (4)
C2—C3—C4—C5	175.8 (3)	C6—N4—C14—C13	146.4 (3)
C6—N3—C5—C4	−0.5 (5)	C11—N4—C14—C13	−54.9 (4)
C7—C4—C5—N3	−5.4 (5)	N5—C13—C14—N4	56.7 (3)
C3—C4—C5—N3	175.5 (3)	C14—C13—N5—C12	−55.3 (3)
C7—N2—C6—N4	175.2 (3)	C14—C13—N5—Co1^iv^	175.84 (18)
C7—N2—C6—N3	−5.8 (5)	C11—C12—N5—C13	53.7 (4)
C14—N4—C6—N2	−9.8 (4)	C11—C12—N5—Co1^iv^	−178.7 (2)
C11—N4—C6—N2	−166.4 (3)	O2—C1—O3—Co1	178.9 (3)
C14—N4—C6—N3	171.1 (3)	C2—C1—O3—Co1	1.0 (4)
C11—N4—C6—N3	14.5 (5)	O1^i^—Co1—O3—C1	179.6 (3)
C5—N3—C6—N2	6.6 (5)	O1—Co1—O3—C1	−0.4 (3)
C5—N3—C6—N4	−174.4 (3)	N5^ii^—Co1—O3—C1	88.4 (3)
C6—N2—C7—N1	−178.7 (3)	N5^iii^—Co1—O3—C1	−91.6 (3)
C6—N2—C7—C4	−1.1 (5)		

Symmetry codes: (i) −*x*+1, −*y*+1, −*z*; (ii) −*x*+2, *y*−1/2, −*z*+1/2; (iii) *x*−1, −*y*+3/2, *z*−1/2; (iv) −*x*+2, *y*+1/2, −*z*+1/2.

### Hydrogen-bond geometry (Å, °)

**Table:**

*D*—H···*A*	*D*—H	H···*A*	*D*···*A*	*D*—H···*A*
N5—H5N···O2^v^	0.90 (1)	2.28 (1)	3.156 (4)	165 (3)

Symmetry codes: (v) −*x*+1, *y*+1/2, −*z*+1/2.
